# Prokaryotic Responses to Ammonium and Organic Carbon Reveal Alternative CO_2_ Fixation Pathways and Importance of Alkaline Phosphatase in the Mesopelagic North Atlantic

**DOI:** 10.3389/fmicb.2016.01670

**Published:** 2016-10-21

**Authors:** Federico Baltar, Daniel Lundin, Joakim Palovaara, Itziar Lekunberri, Thomas Reinthaler, Gerhard J. Herndl, Jarone Pinhassi

**Affiliations:** ^1^Centre for Ecology and Evolution in Microbial Model Systems, EEMiS, Linnaeus UniversityKalmar, Sweden; ^2^Department of Marine Sciences, University of OtagoDunedin, New Zealand; ^3^National Institute of Water and Atmospheric Research (NIWA)/University of Otago Research Centre for OceanographyDunedin, New Zealand; ^4^Division of Bio-Oceanography, Department of Limnology and Oceanography, University of ViennaVienna, Austria; ^5^Institut Català de Recerca de l'AiguaGirona, Spain; ^6^Department of Marine Microbiology and Biogeochemistry, NIOZ Royal Netherlands Institute for Sea Research, Utrecht UniversityDen Burg, Netherlands

**Keywords:** prokaryotic community structure, functional diversity, CO_2_ fixation, alkaline phosphatase, mesopelagic

## Abstract

To decipher the response of mesopelagic prokaryotic communities to input of nutrients, we tracked changes in prokaryotic abundance, extracellular enzymatic activities, heterotrophic production, dark dissolved inorganic carbon (DIC) fixation, community composition (16S rRNA sequencing) and community gene expression (metatranscriptomics) in 3 microcosm experiments with water from the mesopelagic North Atlantic. Responses in 3 different treatments amended with thiosulfate, ammonium or organic matter (i.e., pyruvate plus acetate) were compared to unamended controls. The strongest stimulation was found in the organic matter enrichments, where all measured rates increased >10-fold. Strikingly, in the organic matter treatment, the dark DIC fixation rates—assumed to be related to autotrophic metabolisms—were equally stimulated as all the other heterotrophic-related parameters. This increase in DIC fixation rates was paralleled by an up-regulation of genes involved in DIC assimilation via anaplerotic pathways. Alkaline phosphatase was the metabolic rate most strongly stimulated and its activity seemed to be related to cross-activation by nonpartner histidine kinases, and/or the activation of genes involved in the regulation of elemental balance during catabolic processes. These findings suggest that episodic events such as strong sedimentation of organic matter into the mesopelagic might trigger rapid increases of originally rare members of the prokaryotic community, enhancing heterotrophic and autotrophic carbon uptake rates, ultimately affecting carbon cycling. Our experiments highlight a number of fairly unstudied microbial processes of potential importance in mesopelagic waters that require future attention.

## Introduction

Mesopelagic heterotrophic prokaryotes rely on the organic matter generated by primary production in the sun-lit surface waters. Globally an average of around 70% of the surface water's particulate organic matter is exported into the dark ocean (i.e., outside of the euphotic zone) as sedimenting particles (Arístegui et al., 2005; Buesseler and Boyd, 2009). The majority of particulate organic carbon exported from the euphotic zone is remineralized in the mesopelagic layer, leading to attenuation of flux that is typically parameterized as an exponential decay function of depth (Martin et al., 1987). However, the prokaryotic organic carbon demand (determined as respiration plus biomass production) has been shown to continuously exceed the supply rate of particulate organic carbon in the dark ocean (Reinthaler et al., 2006; Steinberg et al., 2008; Baltar et al., 2009a).

Although, heterotrophic prokaryotes rely on organic carbon, both chemoautotrophs and heterotrophs also incorporate dissolved inorganic carbon (DIC) via a wide range of carboxylation reactions (anaplerotic reactions and the synthesis of fatty acids, nucleotides and amino acids) that form part of their central and peripheral metabolic pathways (Dijkhuizen and Harder, 1984). High dark DIC fixation rates attributed to heterotrophic bacteria have been found in surface waters (Li and Dickie, 1991; Prakash et al., 1991; Li et al., 1993; Markager, 1998; Alonso-Sáez et al., 2010), and an active DIC-fixing prokaryotic community has been observed in the mesopelagic layer of the Atlantic (Herndl et al., 2005; Reinthaler et al., 2010; Baltar et al., 2010b). DIC fixation in the mesopelagic realm has been suggested as a process that could explain this imbalance between the heterotrophic carbon demand and its supply (Baltar et al., 2010b), but the energy source to sustain this DIC fixation remains enigmatic. There is evidence suggesting that ammonium and/or sulfur oxidation could be responsible for sustaining DIC fixation rates (Agogué et al., 2008; Swan et al., 2011). Although, DIC fixation potentially plays a previously unrecognized role in the mesopelagic carbon cycle, a mechanistic understanding of this process in the vast mesopelagic realm remains to be deciphered (Swan et al., 2011; Herndl and Reinthaler, 2013).

Alkaline phosphatase (APase) activity is supposed to be regulated by the concentration of its end product, decreasing with increasing inorganic phosphate concentrations (Chrost, 1990). Since inorganic phosphate is readily available in the deep ocean, APase activity would be expected to be low. However, high APase activities at high Pi concentrations have been found in the deep Indian and Atlantic Ocean (Hoppe and Ullrich, 1999; Tamburini et al., 2002; Baltar et al., 2009b, 2010a, 2013). Thus, although APase appears to play a key role in the dark ocean, understanding is lacking of how APase activity is controlled and what mechanisms are responsible for its regulation in the dark ocean.

During the last decades, marine microbial ecologists have developed and applied molecular biology techniques to uncover the population genetics underlying microbial processes in the sea (DeLong, 2009). Thus, information about the role of different marine prokaryotic groups in processing of organic matter and recycling of elements in the sea is currently accumulating, indicating that different patterns of nutrient cycling can be attributed to differences in the genetic structure of the prokaryotic community. Currently, metatranscriptomics (i.e., analyses of microbial community gene expression through next-generation deep-coverage RNA sequencing) is being used to interrogate marine microbial communities by linking taxonomic structure and function (e.g., Frias-Lopez et al., 2008; Vila-Costa et al., 2010). As a result of this work, our perception of the physiological capabilities of marine prokaryotes in regulating element cycling and fluxes of energy in the sea is changing.

The aim of this study was to investigate potential links between nutrient inputs, bacterioplankton community composition and activity (from gene expression to bulk rates), and analyze microbial processes that may be of potential importance in the dark ocean. We hypothesized that DIC fixation would be stimulated through energy obtained from oxidation of thiosulfate or ammonium, while input of dissolved organic matter would cause a strong activation of heterotrophic activities at the expense of processes like DIC fixation driven by chemolitotrophy. We also hypothesized that APase activities would increase in response to nutrient inputs and that this increase in APase rates would be linked to changes in expression levels of genes involved in the regulation of elemental balance. Thus, we determined changes in prokaryotic diversity by 16S rRNA gene sequencing and heterotrophic and autotrophic activity in response to different organic carbon (pyruvate plus acetate) and inorganic N (ammonium) and S (thiosulfate) compounds in three microcosm experiments with mesopelagic water from the subtropical North Atlantic. In one experiment, we also studied the gene expression by the marine prokaryotic community (metatranscriptomics) in response to those treatments. The combination of 16S rRNA gene pyrosequencing and metabolic rate measurements together with the changes in gene expression allowed us to shed light onto the responses of the prokaryotic community to nutrient pulses in the open ocean twilight zone, from the community composition to the functional response.

## Materials and methods

### Study site and experimental setup

**Figure 1:**
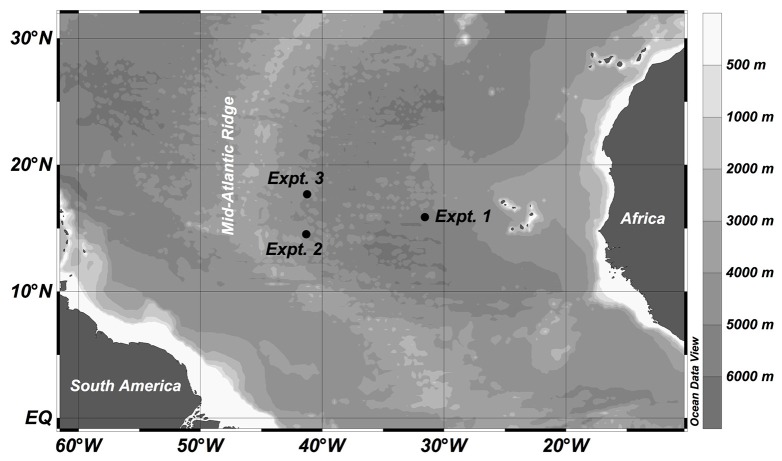
**Locations where upper mesopelagic water was obtained to establish the experiments during the MOCA cruise in 2010**.

Three experiments were conducted with water collected at different sites (Stations 6, 16, and 17) in the subtropical northeast Atlantic Ocean during the MOCA cruise with the RV *Pelagia* in October-November 2010 (Figure 1). St. 6, used in Experiment 1, was located closer to the African shelf region than the other two stations (i.e., Stn 16 for Expt 2 and Stn 17 for Expt 3), which were open ocean stations just east of the Mid-Atlantic ridge. For each experiment, 100–160 l of seawater was collected from the upper mesopelagic zone (320–510 m) using 25 l Niskin bottles mounted on a CTD rosette sampler. The physical, chemical (salinity, temperature, oxygen, inorganic nutrients) and biological (rates, community composition) characteristics of the collected waters were determined to characterize the *in situ* conditions at the sampling locations (Table 1).

**Table 1:** **Mean values of physical, chemical, and biological properties characterizing the waters used for the experiments**.

**Expt**	**Station**	**Date**	**Long (°E)**	**Lat (°N)**	**Depth [m]**	**Theta [°C]**	**Salinity**	**O_2_ [μmol/kg]**	**PO_4_ [μmol/kg]**	**NH_4_ [μmol/kg]**	**NO_3_ [μmol/kg]**	**NO_2_ [μmol/kg]**	**SiO_4_ [μmol/kg]**	**DOC [μmol/kg]**	**PHP [μmol C/m^3^/d]**	**DIC [μmol C/m^3^/d]**	**DIC:PHP**
1	6	10/15/2010	−32.03	15.86	395	10.95	35.33	61.57	1.942	0.07	32.15	0.017	13.2	51.0	0.67	0.48	0.71
2	16	10/25/2010	−41.08	14.66	319	10.88	35.29	94.07	1.770	0.05	28.67	0.012	12.4	55.7	0.53	0.09	0.17
3	17	10/26/2010	−41.05	17.71	510	10.53	35.34	94.20	1.744	0.03	28.78	0.013	13.0	47.6	0.25	0.15	0.61

*Theta, potential temperature; DOC, dissolved organic carbon; PHP, prokaryotic heterotrophic production measured via leucine incorporation; DIC, dissolved inorganic carbon fixation*.

Three different treatments and a control were established after pre-filtering the seawater through 0.8-μm polycarbonate filters in order to study the potential response of prokaryotes, with the methodological caveat of missing prokaryotes attached to bigger particles. Microcosms were amended either with thiosulfate (Na_2_S_2_O_3;_ 100 μM S, final concentration), ammonium (NH_4_Cl; 100 μM N, final concentration) or pyruvate plus acetate (C_3_H_3_NaO_3_ and C_2_H_3_NaO_3_; 100 μM C, final concentration). These organic carbon compounds were selected since previous studies show that they can account for significant portions of the daily uptake of organic carbon by bacteria (Obernosterer et al., 1999; Ho et al., 2002; Gómez-Consarnau et al., 2012) and be a major component to dissolved organic carbon in seawater (Aluwihare et al., 1997). The control was not supplemented with any inorganic or organic nutrients. We used thiosulfate and ammonium since sulfur and inorganic nitrogen compounds are the two main proposed sources of energy for DIC fixation in the deep ocean (Swan et al., 2011). These concentrations of organic carbon were based on previous research showing concentrations of DOC ranging from 11,700 μM in marine aggregates and up to 150 μM in bulk water around aggregates (Herndl, 1992; Alldredge, 2000; Simon et al., 2002). The concentration we selected was similar to concentrations (328 μM) used in previous community transcriptomic response experiments (McCarren et al., 2010). The concentrations of ammonium and thiosulfate were the same as for organic carbon to allow for comparison in a similar magnitude range. Nevertheless, the concentration of ammonium ranges between ranges between 1 and 483 μM in marine snow (Shanks and Trent, 1979).

All treatments and the control were established in duplicate 20 L acid-washed plastic containers and held in the dark at in situ temperature for 110–140 h. Prokaryotic abundance (PA) was sampled daily, while prokaryotic heterotrophic production (PHP) and extracellular enzymatic activity (EEA) were measured 24 h after setting up the experiment and at the end of the experiments. Dissolved inorganic carbon (DIC) fixation was measured only at the end of the incubations. Also at the end of the experiments, DNA samples were taken for later 16S rRNA tag pyrosequencing (Expt 1 and 3) and RNA extraction for metatranscriptomic analysis (Expt 2) as described below. We did not do 16S rRNA pyrosequencing of Expt 2 because we used all the biomass collected (filters) to extract RNA to ensure we had enough RNA for the metatranscriptomic analysis. From the thiosulfate treatment of Expt 1 we obtained only one sample for DNA analysis but no metabolic rate measurements.

### Prokaryotic abundance (PA)

Picoplankton were enumerated using flow cytometry on a FACSCalibur (Becton-Dickinson) with a laser emitting light at 488 nm. Samples (1.5 ml) were fixed with paraformaldehyde (1% final concentration), incubated at 4°C for 15–30 min and then stored frozen in liquid nitrogen until analysis. Prior to enumerating the cells by flow cytometry and after thawing to room temperature, 200 μl samples were stained with a DMSO-diluted SYTO-13 (Molecular Probes) stock (10:1) at 2.5 μM final concentration. Prokaryotes were identified by their signatures in a plot of side scatter vs. green fluorescence (FL1). High and low nucleic acid-content cells (HNA and LNA, respectively) were separated in the scatter plot of SSC-FL1 (Gasol et al., 1999). HNA cells exhibited higher FL1 than LNA cells.

### Prokaryotic heterotrophic production (PHP)

Bulk PHP was measured by incubating triplicate 10–40 ml samples and formaldehyde-killed blanks (2% final concentration) with 10 nM [^3^H]-leucine (final concentration, specific activity 140 Ci mmol^−1^; PerkinElmer) in temperature-controlled incubators in the dark at *in situ* temperature for 3–24 h. Incubations were terminated by adding formaldehyde (2% final concentration) before filtering the samples and the blanks through 0.2-μm polycarbonate filters (25 mm filter diameter; Millipore). Subsequently, the filters were rinsed three times with 5% ice-cold trichloroacetic acid, dried, and placed in scintillation vials. Scintillation cocktail (8 ml Canberra-Packard Filter Count) was added, and after 18 h, counted in a liquid scintillation counter (Tri-Carb 3100TR, Perkin Elmer). The mean disintegrations per minute (DPM) of the formaldehyde-fixed blanks were subtracted from the mean DPM of the respective samples, and the resulting DPM converted into leucine incorporation rates. Prokaryotic carbon biomass production was estimated using a conservative theoretical conversion factor of 1.55 kg C mol^−1^ Leu assuming no isotope dilution (Kirchman and Ducklow, 1993).

### Dissolved inorganic carbon (DIC) fixation

DIC fixation was measured as previously described (Reinthaler et al., 2010). Briefly, 60 μCi of [^14^C]-bicarbonate was added to 40 ml seawater samples. Triplicate samples and formaldehyde-fixed blanks were incubated in the dark at in situ temperature for 24–72 h. Incubations were terminated by the adding formaldehyde (2% final concentration) to the samples, filtering them onto 0.2-μm polycarbonate filters and followed by a rinse with 10 ml of ultra-filtered seawater (<30 kDa). Thereafter, the filters were exposed to a fume of concentrated HCl for 12 h, transferred into scintillation vials and after adding 8 ml scintillation cocktail (Canberra-Packard, Filter Count) incubated for 18 h. The samples were counted for 10 min. The resulting mean DPM of the samples were corrected for the mean DPM of the blanks and converted into DIC fixed over time by correcting for the natural DIC concentrations measured by continuous flow analysis (Stoll et al., 2001).

### Measurements of prokaryotic extracellular enzymatic activity (EEA)

The hydrolysis of the fluorogenic substrate analogs 4-methylcoumarinyl-7-amide (MCA)-L-leucine-7-amido-4-methylcoumarin and 4-methylumbelliferyl (MUF)-phosphate was measured to estimate the potential hydrolytic activity of leucine aminopeptidase (LAPase) and alkaline phosphatase (APase), respectively (Hoppe, 1983). All chemicals were obtained from Sigma. The procedure was followed as described previously (Baltar et al., 2009b, 2010a). Briefly, EEA was determined after substrate addition and incubation using a spectrofluorometer (RF-1501, Shimadzu) at an excitation and emission wavelengths of 365 and 445 nm, respectively. Samples (3 ml) were incubated in the dark at *in situ* temperature for 24–48 h. The linearity of the increase in fluorescence over time was checked on sets of samples incubated for 24–48 h, resulting in the same hydrolytic rates h^−1^. Subsamples without substrate additions served as blanks to determine the background fluorescence of the samples. Previous experiments showed that abiotic hydrolysis of the substrates is insignificant (data not shown). The fluorescence obtained at the beginning and the end of the incubation was corrected for the corresponding blank. This increase in fluorescence over time was transformed into hydrolysis rates using standard curves established with different concentrations of the fluorochromes MUF and MCA added to 0.2 μm filtered sample water. Final substrate concentrations of 100 μmol l^−1^ for APase and 500 μmol l^−1^ for LAPase were used. These concentrations have been previously determined as saturating substrate concentrations, i.e., resulting in maximum hydrolysis rates. Consequently, the EEAs given throughout the paper represent potential hydrolysis rates.

### DNA sample collection and extraction

To collect the microbial DNA, 7–9 l from experiment 1 and 3 were filtered through a 47 mm 0.2-μm pore-size polycarbonate filter (Poretics, Osmonics Inc.), immediately transferred into cryovials containing TE buffer (10 mM Tris, 1 mM EDTA, pH = 8.0) and frozen at −80°C until further processing. A combined treatment with enzymes (lysozyme, proteinase K) and enzyme/phenol-chloroform was used to extract the DNA as described previously (Riemann et al., 2000) but with a 30-min lysozyme digestion at 37°C and an overnight proteinase K digestion at 55°C (Boström et al., 2004). DNA was quantified using PicoGreen (Molecular Probes).

### PCR and sequencing preparation

Partial bacterial 16S rRNA genes were amplified for pyrosequencing using a primer cocktail containing the degenerate primers 530F (5′-GTGCCAGCMGCNGCGGTA-3′; Dowd et al., 2008) with TA added at the 3-prime end to increase specificity, and 1061R (5′-CRRCACGAGCTGACGAC-3′; Dowd et al., 2008) labeled with specific hexamers to differentiate each sample (Sjöstedt et al., 2012). The PCR products were excised from a 2% agarose gel, purified with a gel extraction kit (QIAquick, Qiagen) and concentrated with a PCR purification kit (QIAquick, Qiagen). Linkage of adaptors and pyrosequencing on the 454 GS FLX system using TITANIUM chemistry (Roche Applied Science) were performed at LGC Genomics (Germany) according to the manufacturer's instructions.

### 16S rRNA gene sequence analysis

From the 17 samples collected, a total of 346,236 sequences (average length 292 bp) were obtained and analyzed following the approach described previously (Fierer et al., 2008) using the Quantitative Insights Into Microbial Ecology (QIIME) pipeline (http://qiime.org). Low quality sequences were removed (sequences < 200 bp in length). The average remaining sequences after the bioinformatics treatments ranged between 39,001 and 1466 reads per sample (average = 20,047), with all rarefaction curves indicating saturation. Denoising was done via the n3phele cloud (http://www.n3phele.com) using the QIIME Denoiser algorithm (v. 0.91). Singletons were not included in any further analyses. Similar sequences were binned into operational taxonomic units (OTUs) using UCLUST (Edgar, 2010) with a minimum pairwise identity of 97%. Representative sequences for each OTU were aligned with PyNAST, the taxonomic identity of each phylotype determined using the RDP Classifier (Wang et al., 2007), and a tree built using FastTree (Price et al., 2009). A bootstrapped and jackknifed hierarchical cluster analysis was done using the UPGMA (Unweighted Pair Group Method with Arithmetic mean) clustering method within QIIME, generating a bootstrapped tree with colored internal nodes indicating the level of support (red for 75–100% support, yellow for 50–75%, green for 25–50%, and blue for <25% support). We also determined the level of several alpha diversity metrics (i.e., Margalef, Shannon and Simpson indexes) in our samples using the default settings in QIIME. Data was uploaded to ENA (accession number PRJEB15590).

### Metatranscriptomics

Samples for community-wide mRNA analyses were collected by filtering 15–20 L from the treatment and the controls bottles. Filters were put in 15 ml Falcon tubes containing 2 ml RLT buffer (containing 10 μl beta-mercaptoethanol per milliliter) and flash frozen in liquid nitrogen before storage at −80°C. Messenger RNA was extracted essentially according to the protocol of Poretsky et al. (2009), using the RNEasy kit (Qiagen). Genomic DNA was removed using the TURBO DNA-free kit (Ambion) and ribosomal RNA was removed enzymatically using mRNA-ONLY prokaryotic mRNA isolation kit (Epicenter Biotechnologies) and MICROBExpress (Ambion) according to the manufacturers' recommendations. Bacterial mRNA was further enriched using MICROBEnrich (Ambion). cDNA synthesis from mRNA was carried out using AMBION message Amplification II-Bacteria kit (Ambion). The MessageAmp II-Bacteria Kit (Ambion) was used to linearly amplify RNA. cDNAs from each sample were sequenced using the HiSeq 2000 sequencing system (Illumina) at SciLifeLab Stockholm.

Sequence reads were processed as previously described in detail (Bunse et al., 2016). Briefly, RNA sequences were quality checked, trimmed, and filtered against the Silva database of small and large subunit ribosomal RNA using the filtering mode of the Erne mapper40 with default parameters. Subsequently, all samples were individually assembled using the Ray and Velvet. Open reading frames (ORFs) were called on resulting contigs using FragGeneScan. The resulting ORFs were clustered before annotation to remove duplicates using Usearch. Subsequently, the resulting ORFs were annotated using BLAST against the M5NR SEED, KEGG, and RefSeq databases, followed by annotation using in-house developed scripts (available at https://github.com/erikrikarddaniel/environmentmicrobedb-tools). Sequences with identical functional annotations were summed to calculate abundances of transcripts of “genes” in the community. For comparison of gene expression levels between samples, relative transcript abundances for each gene were calculated by dividing the number of transcript reads per gene by the number of annotated reads per sample. These relative transcript abundances are presented as counts per million (CPM). Data was uploaded to ENA (accession number PRJEB3383).

### Statistical analyses

To compare the different sets of samples, we carried out analyses of variance (ANOVA) followed by Tukey's HSD test to compare the group means after log-transformation of the data to attain normality using the JMP Statistical Software (SAS Institute Inc, Cary, NC). Normality was checked with a Shapiro-test. Tukey's HSD considers multiple comparisons and corrects for type I errors.

## Results

### Oceanographic settings

Stations 16 and 17 (where water for Expt 2 and 3 was collected) shared similar characteristics with generally lower concentrations in inorganic nutrients and metabolic rates but higher oxygen concentrations than Stn 6 (water collected for Expt 1; Table 1). The DOC concentrations were also similar among the three stations, ranging between 47.6 and 55.7 μmol kg^−1^.

### Temporal dynamics of prokaryotic abundance and metabolic rates

**Figure 2:**
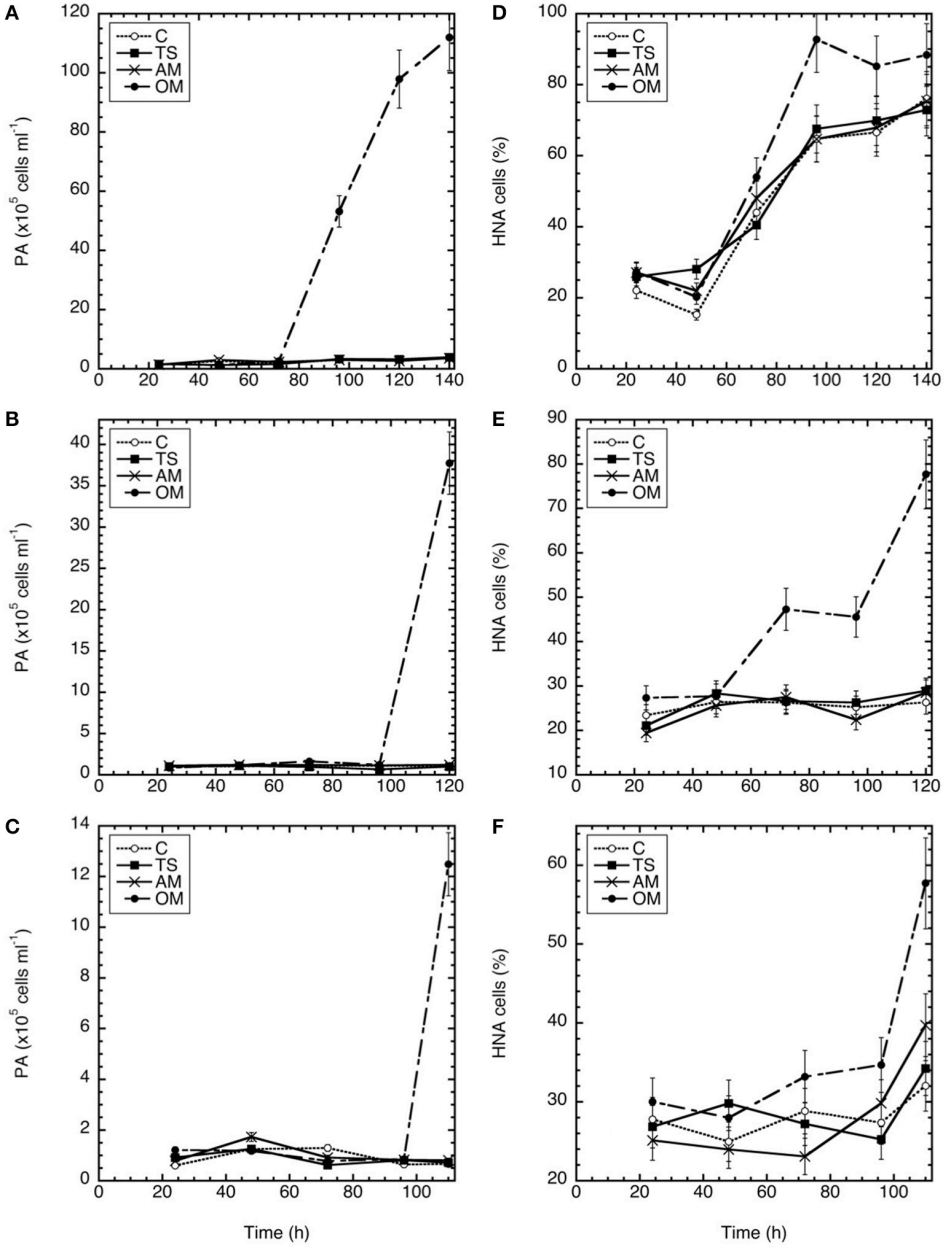
**Temporal variation in prokaryotic abundance (A–C) and percentage of high nucleic acid content cells (D–F) in Expt. 1 (A,D), Expt. 2 (B,E), and Expt. 3 (C,F)**. C, control; TS, thiosulfate; AM, ammonium; OM, pyruvate + acetate.

Total prokaryotic abundance (PA) showed a similar temporal pattern in all three experiments (Figures 2A–C), and remained stable until the final sampling point in the thiosulfate and ammonium amended microcosms and the controls. Only in the organic matter treatment, PA showed pronounced increases between 12 and 112 × 10^5^ cells ml^−1^ toward the end of the experiments, with higher peak abundances in Expt 1.

In all three experiments, high nucleic acid (HNA) content cells initially constituted 20–30% of the prokaryote abundance (Figures 2D–F). Similar to PA, the highest percentage of HNA prokaryotes was detected at the end of the experiments (93, 78, and 58% for Expt 1, 2, and 3, respectively). The %HNA increased in all treatments in Exp 1 and 3, but only in the OM treatment in Exp 2. In Exp 3 the %HNA response was later than the other experiments. Among the three amendments, the highest percentage of HNA was found in the organic matter amendment (Tukey-HSD, α < 0.05) (Figures 2D–F).

Prokaryotic heterotrophic production (PHP) and extracellular enzymatic activity (EEA) generally increased over the course of the experiments (Table 2). At the end of the experiments, all rates were 1–2 orders of magnitude higher than the initial rates in all three treatments and also in the controls. Generally, PHP was the metabolic rate showing the strongest enhancements when comparing the different subsampling times, particularly in the organic matter treatment (up to 3 orders of magnitude). Leucine aminopeptidase (LAPase) and alkaline phosphatase (APase) also increased with time, but to a lower extent than PHP in the shorter experiments (i.e., Expt 2 and 3).

**Table 2:** **Prokaryotic abundance, bulk, and cell-specific metabolic rates obtained 24 h after starting the experiments and at the end of the experiments**.

**Time (h)**	**Expt**	**Treat**	**PA**	**DIC**	**PHP**	**LAPase**	**APase**	**Cell-spec PHP**	**Cell-spec LAPase**	**Cell-spec APase**	**DIC:PHP**
24	1	C	1.4	n.d.	0.6	0.9	0.4	0.004	6.1	2.8	n.d.
24	1	TS	1.6	n.d.	0.9	0.8	0.4	0.006	4.9	2.4	n.d.
24	1	AM	1.5	n.d.	0.8	0.7	0.4	0.006	5.0	2.8	n.d.
24	1	OM	1.7	n.d.	1.7	0.5	0.9	0.010	2.8	5.1	n.d.
24	2	C	1.0	n.d.	1.7	0.8	0.6	0.017	8.2	6.0	n.d.
24	2	TS	1.0	n.d.	1.1	0.8	0.5	0.010	7.6	5.3	n.d.
24	2	AM	1.1	n.d.	0.9	0.8	0.6	0.008	7.3	5.5	n.d.
24	2	OM	0.9	n.d.	1.2	6.5	41.2	0.014	76.3	482.8	n.d.
24	3	C	0.6	n.d.	0.8	1.0	0.3	0.013	17.0	5.3	n.d.
24	3	TS	0.9	n.d.	0.7	n.d.	n.d.	0.008	n.d.	n.d.	n.d.
24	3	AM	0.8	n.d.	0.5	n.d.	n.d.	0.006	n.d.	n.d.	n.d.
24	3	OM	1.2	n.d.	2.5	n.d.	n.d.	0.021	n.d.	n.d.	n.d.
140	1	C	3.7	45.9	37.1	8.2	8.3	0.102	22.4	22.7	1.2
140	1	TS	3.7	n.d.	n.d.	n.d.	n.d.	n.d.	n.d.	n.d.	n.d.
140	1	AM	3.6	111.1	31.8	11.3	11.6	0.089	31.7	32.4	3.5
140	1	OM	112.0	240.6	162.5	70.0	134.8	0.015	6.3	12.0	1.5
120	2	C	1.1	15.6	20.8	1.3	0.3	0.193	12.4	3.0	0.7
120	2	TS	1.0	13.5	11.4	1.1	0.3	0.115	11.3	2.7	1.2
120	2	AM	1.2	32.9	16.2	1.4	0.9	0.134	11.3	7.7	2.0
120	2	OM	37.7	987.6	2033.2	112.2	239.6	0.539	29.7	63.5	0.5
110	3	C	0.7	9.8	33.0	1.1	1.0	0.497	16.9	14.8	0.3
110	3	TS	0.7	11.4	40.4	1.4	0.7	0.565	19.9	9.2	0.3
110	3	AM	0.8	24.3	45.5	1.9	0.9	0.560	23.4	11.4	0.5
110	3	OM	12.5	185.0	1747.8	48.1	128.7	1.400	38.5	103.1	0.1

*Expt, experiment; Treat, treatment; PA, prokaryotic abundance (x10^5^ cells ml^−1^); DIC, dissolved inorganic carbon fixation (μmol C m^−3^d^−1^); PHP, prokaryotic heterotrophic production (μmol C m^−3^d^−1^); LAPase, leucine aminopeptidase (nmol l^−1^ h^−1^); APase, alkaline phosphatase (nmol l^−1^ h^−1^); Cell-spec PHP, cell specific prokaryotic heterotrophic production (fmol C cell^−1^ d^−1^); Cell-spec leucine aminopeptidase (amol cell^−1^ h^−1^), Cell-spec APase, alkaline phosphatase (amol cell^−1^ h^−1^). C, unamended control; TS, thiosulfate amendment; AM, ammonium; OM, acetate + pyruvate. n.d., not determined*.

### Treatments effects on prokaryotic abundance and metabolic rates

**Figure 3:**
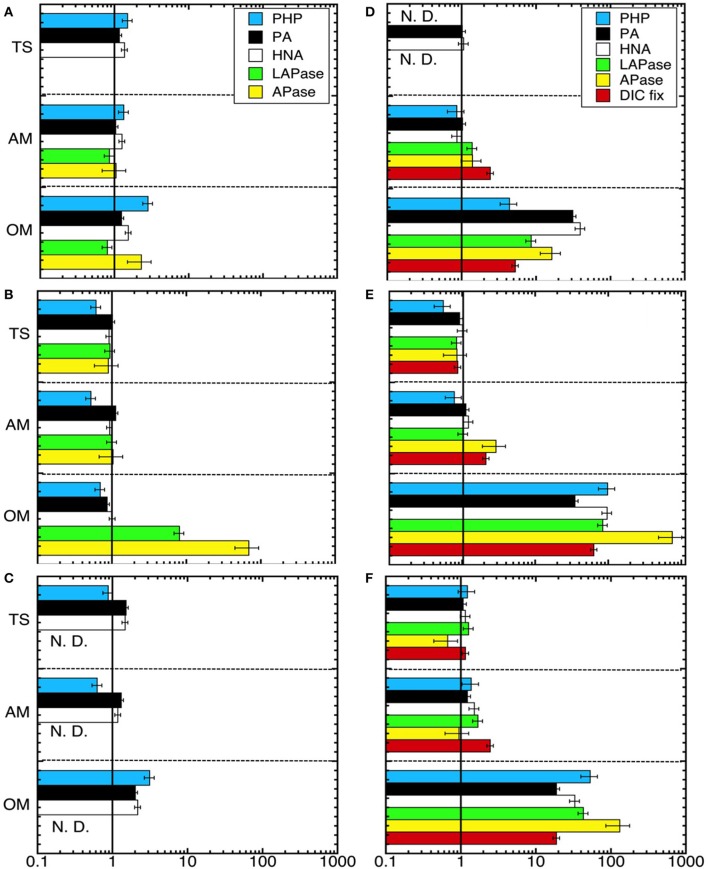
**Ratio of the bulk metabolic rates obtained in the different treatments divided by the rates obtained in the control 24 h after (A–C) the amendment and at the end (D–F) of Expt. 1 (140 h) (A,D), Expt. 2 (120 h) (B,E), and Expt. 3 (110 h) (C,F)**. Error bars show the propagated errors for each parameter for the treatment and control. The line *x* = 1 indicates no stimulation of rates due to specific treatment effects. Treatments within an experiment are separated by hashed horizontal lines. PHP, prokaryotic heterotrophic production; PA, prokaryotic abundance; HNA, abundance of high nucleic acid content cells; LAPase, leucine aminopeptidase; APase, alkaline phosphatase; DIC, dissolved inorganic carbon fixation. TS, thiosulfate; AM, ammonium; OM. pyruvate + acetate.

As a measure of the treatment effect, we computed the ratio between the rates obtained in each treatment divided by the corresponding rates measured in the unamended control (Figure 3). After 24 h of incubation no effects were found in incubations with ammonium or thiosulfate additions (ANOVA *p* > 0.05), however, the organic matter amendment exhibited increased PHP (in Expt 1 and Expt 3) and EEA (in Expt 2) (Tukey-HSD, α < 0.05; Figures 3A–C). The strongest treatment effects were found at the end of the experiments in the organic matter amended microcosms (Figures 3D–F), where PHP, EEA and DIC fixation rates increased by 2–3 orders of magnitude as compared to the control. This enhanced microbial activity in the organic matter treatment was also reflected in the high PA, particularly due to the contribution of HNA cells (Figure 2). Among the measured rates, APase was most strongly stimulated in the experiments (Figures 3D–F) increasing up to 750 times as compared to the control in the organic matter treatment of Expt 2 (Figure 3E). Over the time course of the experiments, thiosulfate additions exhibited no discernible effect on rate measurements as compared to the control (ANOVA *p* < 0.05). In the ammonium amendment, dark DIC fixation rates increased by a factor of 2–3 (Tukey-HSD, α < 0.05; Table 2, Figures 3D–F). In contrast to the organic matter amendment, the increase in DIC fixation rates found in the ammonium treatment did not coincide with an increase in PA.

### Treatments effects on prokaryotic community structure

**Figure 4:**
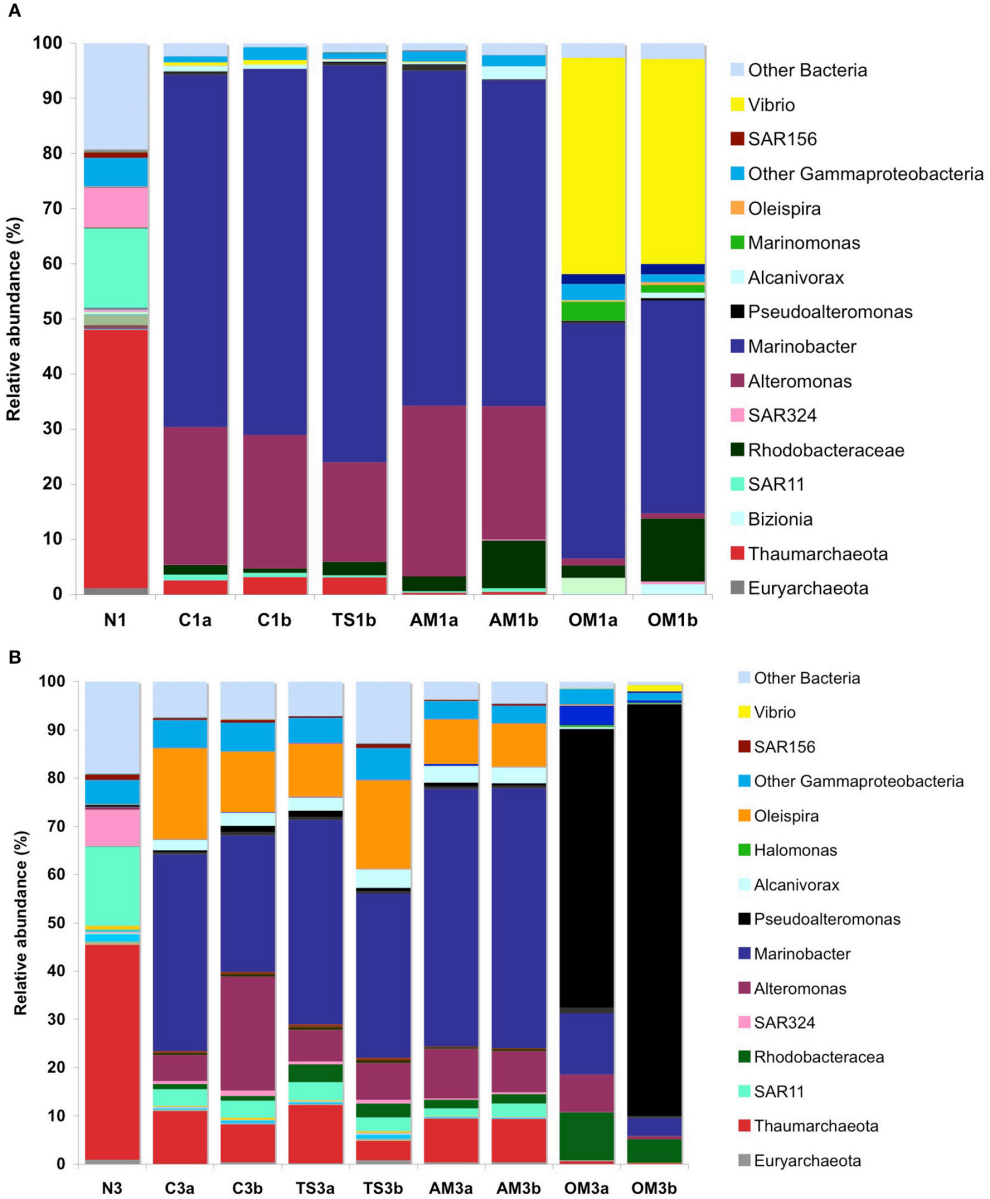
**Composition of prokaryotic assemblages in the enrichment cultures down to the genera level**. Percentage of the relative abundance at the end of Exp. 1 **(A)** and 3 **(B)**, in duplicate carboys (a,b). N, natural waters; C, unamended control; TS, thiosulfate; AM, ammonium; OM, pyruvate + acetate. Example: “AM1a” and “AM1b” are duplicate carboys of the ammonium treatment of Expt. 1. Note that some sequences could not be resolved down to the general level, but the deepest resolved taxonomic level was included in this plot (e.g., other Gammaproteobacteria, Thaumarchaeota, SAR11, SAR324, etc.).

Despite the large geographic distance (and different biogeochemical characteristics) between Stn 6 at the continental shelf and Stn 17 at the mid-Atlantic Ridge (Figure 1), both locations shared a similar *in situ* prokaryotic assemblage (Figure 4). However, the changes in prokaryotic community structure during the course of the two experiments differed distinctly.

**Figure 5:**
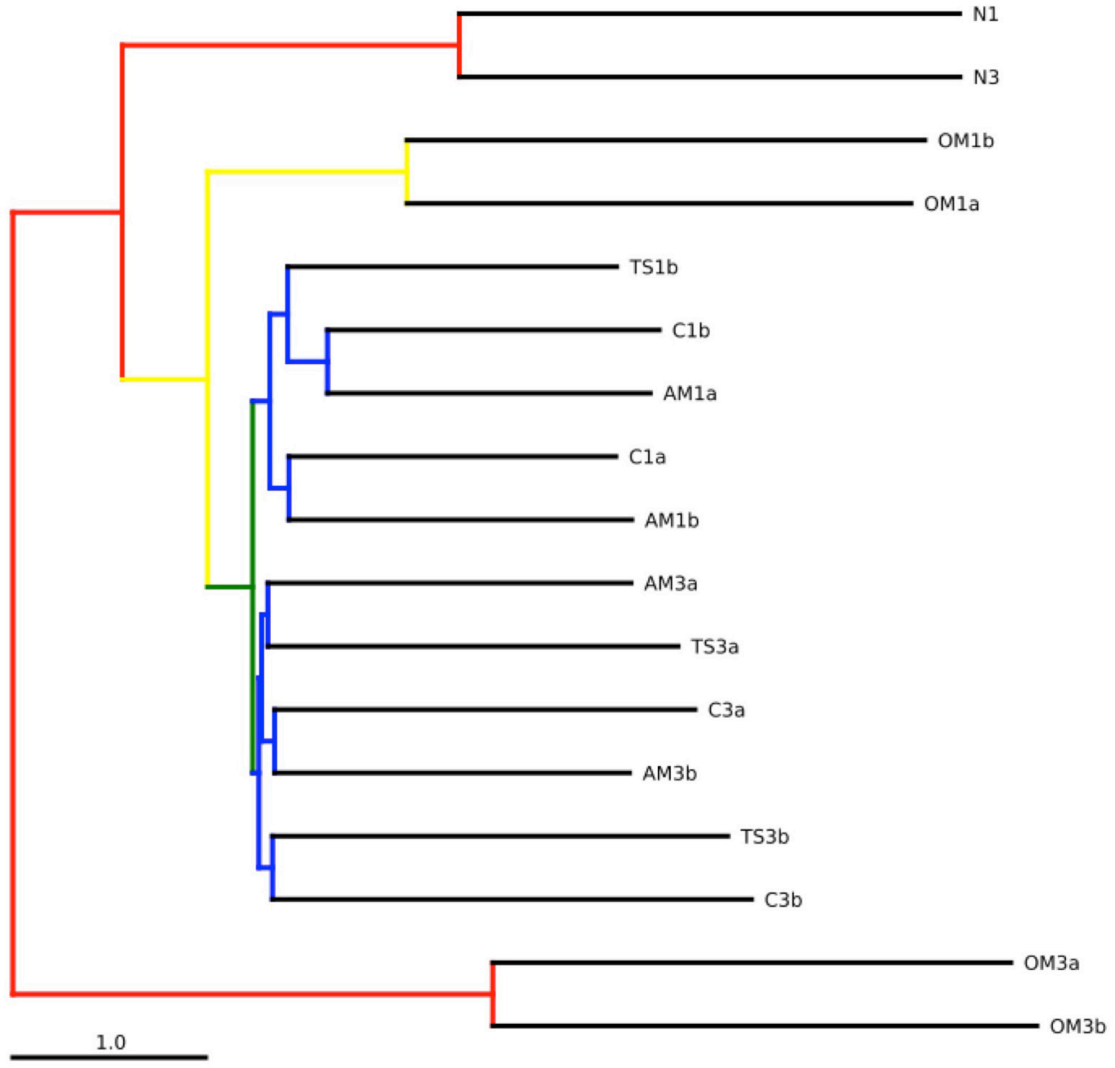
**Bootstrapped jackknifed hierarchical clustering analysis using UPGMA (Unweighted Pair Group Method with Arithmetic mean)**. Internal nodes colored indicating the level of support (red: 75–100%, yellow: 50–75%, green: 25–50%, blue: <25%). N, natural waters; C, unamended control; TS, thiosulfate; AM, ammonium; OM, pyruvate + acetate.

The jackknifed UPGMA showed that the natural prokaryotic communities used for the experiments were similar (Figure 5). During the course of the experiments, the community composition diverged from the initial communities and between treatments (Figure 5). The organic matter treatment exhibited the most pronounced changes in community compositions among all the treatments. The communities from the thiosulfate and ammonium treatments and the controls of each experiment formed two subclusters, one for each experiment.

The observed number of distinct populations (i.e., operational taxonomic units [OTUs] defined by sequence clustering at 97% 16S rRNA gene sequence identity), after removing all the singletons, was the same (207 OTUs) in the controls of both experiments (Table 3). This number was reduced by 73% at the end of Expt 1 (e.g., 55–57 OTUs in the control) and by 20–25% in the Expt 3 (e.g., 156–164 OTUs in the control). A strong treatment effect on prokaryotic richness was found in the organic matter treatment, where the number of observed OTUs was ~50 and 70% lower than in the unamended controls at the end of Expt 1 (56 ± 1 and 26 ± 11 [mean ± SD] for the control and the organic matter treatment, respectively) and Expt 3 (160 ± 6 in the control and 48 ± 17 in the organic matter treatment), respectively. This overall pattern of richness reduction during the incubation due organic matter amendment was reflected by the Margalef index (Table 3). Also, the Shannon and Simpson indexes indicated a substantial decrease in diversity from the original prokaryotic communities to that obtained at the end of both experiments (Table 3). A lower diversity was found in the organic matter treatment as compared to the other amendments at the end of Expt 3, but not for Expt 1.

**Table 3:** **Number of observed operational taxonomic units (OTUs) and alpha-diversity indexes for the natural waters and at the end of Expt. 1 and 3**.

**Treatment**	**Observed OTUs**	**Margalef**	**Shannon**	**Simpson**
N1	207	29.3	5.35	0.93
C1b	55	7.7	2.7	0.75
C1a	57	7.9	2.76	0.75
TS1b	34	5.27	2.34	0.65
AM1a	48	6.69	2.54	0.72
AM1b	37	5.75	2.85	0.75
OM1b	19	3.75	3.05	0.82
OM1a	34	5.1	2.96	0.79
N3	207	29.3	5.4	0.93
C3a	156	22	4.58	0.88
C3b	164	23.2	5.02	0.92
TS3a	158	22.3	4.64	0.88
TS3b	198	28	5.17	0.91
AM3b	130	18.4	4.04	0.81
AM3a	112	15.8	3.93	0.83
OM3a	60	8.4	3.17	0.79
OM3b	36	5	2	0.63

*C, unamended control; AM, ammonium; OM, acetate + pyruvate; TS, thiosulfate; N, natural original waters. a,b–denote replicate treatments*.

In Expt 1, several prokaryotic populations decreased to below the detection level in the control during the course of the experiment, including taxa belonging to Euryarchaeota and Actinobacteria, some members of the Bacteroidetes (Cryomorphaceae and Tenacibaculum, and the Sphingobacteria) and some Proteobacteria (all Deltaproteobacteria and some Gammaproteobacteria) (Table S2). In contrast, other Bacteria in the control increased in their relative abundance as compared to the original community, including one family within the Alphaproteobacteria (Rhodobacteraceae) and three different Gammaproteobacteria genera (two from the order Alteromonodales [*Alteromonas* and *Marinobacter*] and one from the order Oceanospirillales [*Alcanivorax*]).

Besides the changes in the control described above, specific treatment effects were also found. In Expt 1, the relative abundance of some bacterial genera changed in the organic matter treatment as compared to the control, such as for Bacteroidetes (the Flavobacteria genus *Bizionia*) and Gammaproteobacteria (one genus from the order Alteromonodales [*Pseudoalteromonas*], two genera from the order Oceanospirillales [*Marinomonas* and *Oleispira*] and the genus *Vibrio*) (Table S2). Particularly remarkable was the increase of an OTU assigned to the genus *Vibrio*, from a relative abundance of 0.03% in the natural waters to 37–39% at the end of the incubation in the organic matter treatment. The Alphaproteobacteria family Rhodobacteraceae increased not only in the organic matter treatment but also in all other treatments as compared to the control. Remarkably, all the groups that increased in the amendments belonged to the rare biosphere fraction, (sensu Sogin et al., 2006 i.e., representing a relative OTU abundance of <1% in the *in situ* community).

In Expt 3, a similar pattern as in Expt 1 was found in the community composition. Many of the groups that disappeared completely at the end of Expt 1, however, were still detected at the end of Expt 3, albeit greatly reduced in their relative abundance, particularly in the organic matter treatment as compared to the other treatments (Table S3). This was particularly evident for the Archaea and Acidobacteria. Other taxa disappeared completely at the end of Expt 3, including several Planctomycetes and Gammaproteobacteria genera. Also the groups increasing in relative abundance during the course of the Expt 3 were similar to those in Expt 1, and consisted of Rhodobacteraceae, Alteromonodales and Oceanospirillales. Among Alteromonodales there were particularly strong responses of Alteromonadaceae, Colwelliaceae and Pseudoaltermonadaceae, and Oceanospirillales with the genera *Alcanivorax, Marinomonas*, and *Oleispira*.

The response for a given treatment between experiments was not identical. Although, the groups increasing in relative abundance due to treatment effects in Expt 3 were similar to those in Expt 1, another genus of Oceanospirillales (*Halomonas*) also increased in the organic matter treatment in Expt 3. In contrast to Expt 1, no Bacteroidetes increased in any of the treatments, possibly because *Bizionia*, the only Bacteroidetes member that increased in relative abundance in Expt 1, was not detected in the original waters of Expt 3. As in Expt 1, Rhodobacteraceae was the only taxon increasing in relative abundance in all treatments as compared to the control in Expt 3. In contrast to Expt 1 where *Vibrio* increased most pronouncedly, *Pseudoalteromonas* was the group showing the highest increase during the course of Expt 3 from a relative abundance of 0.4 to 58–85%. As in Expt 1, all groups that responded to the amendments with an increase in abundance belonged to the rare biosphere in the original waters. This suggests that small variations in the relative abundance or proportion of rare members in the *in situ* communities and slight differences in environmental conditions might affect which are the specific members becoming dominant in response to the same treatment at the end of the experiment.

### Treatments effects on microbial gene expression

Sequencing of messenger RNA (mRNA) genes expressed by the bacterial assemblages in the enrichment cultures showed that the overall dominant functional categories included the clustering-based subsystem, carbohydrates, protein metabolism (and amino acids and derivatives) and fatty acids, lipids, and isoprenoids (Table S1). The transcription of the nitrogen metabolism was 2–3 times lower in ammonium amendment as compared to the other treatments. As observed in the metabolic rates, the relative abundance of the transcripts of most categories increased from the thiosulfate to the ammonium and organic matter amendment (Table S1).

**Figure 6:**
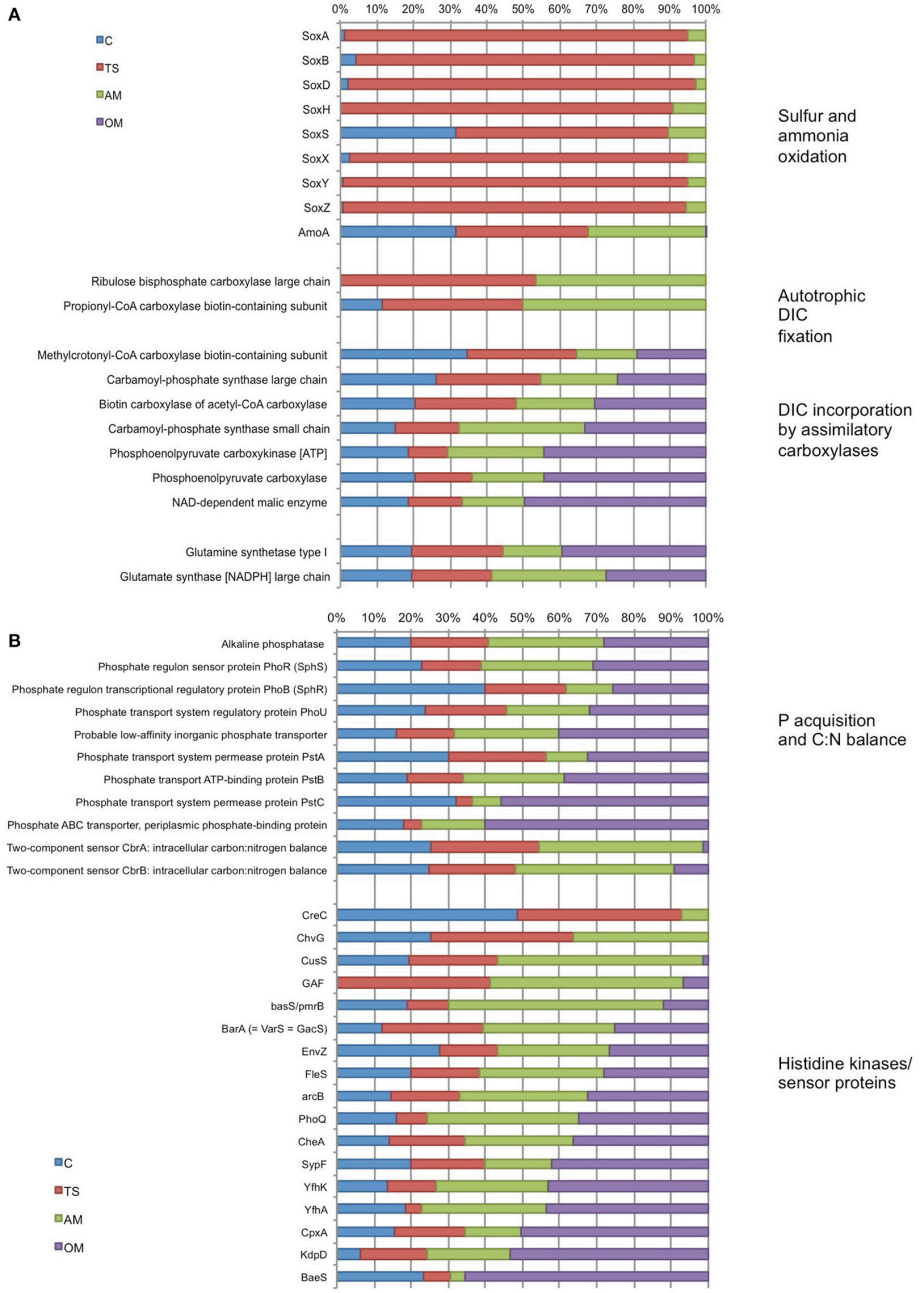
**Proportion (%) of transcripts retrieved at the end of Expt. 2 (120 h) in the different treatments involved in (A) sulfur (sox) and ammonia oxidation, autotrophic DIC fixation and DIC incorporation by assimilatory carboxylases, and in (B) phosphate acquisition, intracellular C:N balance and histidine kinase/sensor proteins**. Numbers on the right are the summed relative abundances in counts per million (CPM) in all treatments and control for each gene. C, unamended control; TS, thiosulfate; AM, ammonium; OM, pyruvate + acetate. *sox*, sulfur oxidation protein Sox; *amoA*, ammonia monooxygenase AmoA; *creC*, two-component response regulator CreC; *chvG*, sensor histidine kinase ChvG; *cusS*, copper sensory histidine kinase CusS; GAF sensor signal transduction histidine kinase; *basS/pmrB*, Sensor protein basS/pmrB; *barA*, sensory histidine kinase BarA (= VarS = GacS); *envZ*, osmolarity sensory histidine kinase EnvZ; *fleS*, flagellar sensor histidine kinase FleS; *arcB*, aerobic respiration control sensor protein ArcB; *phoQ*, sensor protein PhoQ; *cheA, s*ignal transduction histidine kinase CheA; *sypF*, signal transduction histidine kinase SypF; *yfhK*, putative sensor-like histidine kinase YfhK; *yfhA*, putative sensor-like histidine kinase YfhA; *cpxA*, copper sensory histidine kinase CpxA; *kdpD*, osmosensitive K+ channel histidine kinase KdpD; *baeS*, sensory histidine kinase BaeS.

The transcription of *sox* (sulfur oxidation) genes was highly prominent in the thiosulfate treatment (58–96% of the collective *sox* transcripts), whereas the expression of these genes in the ammonium treatment was low (<12% of *sox* transcripts) and remained undetected in the organic matter treatment (Figure 6A). In the unamended controls, *sox* genes were detectable at very low levels (except for *soxS*). The expression of *amoA* (ammonia monoxygenase) was similar in the control, the thiosulfate and ammonium treatments but virtually undetectable in the organic matter treatment. Almost all the transcripts of the genes related to autotrophic DIC fixation by the 3-hydroxypropionate/4-hydroxybutyrate cycle (i.e., propionyl-CoA carboxylase) and the Calvin-Benson-Bassham Cycle (i.e., RuBisCO) were found in low relative abundance in the ammonium and thiosulfate treatment (Figure 6A). Some of the transcripts of genes involved in anaplerotic DIC assimilation (methylcrotonyl-CoA carboxylase, carbomoyl-phosphate synthase and acetyl-CoA carboxylase) were roughly equally abundant in all the treatments, whereas others (phosphoenolpyruvate carboxykinase, phosphoenolpyruvate carboxylase and malic enzyme) were higher in the organic matter treatment (Figure 6A).

Overall, the organic matter and ammonium treatments accounted for the majority (on average 60%) of the collective transcripts related to inorganic phosphorus acquisition (Figure 6B). The *pstABCS* gene system (ABC transporter) accounted for a major portion of the expressed genes for phosphate uptake, particularly in the organic matter treatment where the genes encoding the PstC and PstS subunits each reached nearly 60% of the collective transcripts. Both genes of the two-component CbrA-CbrB system, involved in the control of the utilization of multiple carbon and nitrogen sources in relation to intracellular C:N balance, reached highest abundance in the ammonium treatment (~45% of *cbr* transcripts). Although, they exhibited fairly high relative abundances also in the control and the thiosulfate treatment, *cbrAB* relative abundances were very low in the organic matter treatment. The proportion of transcripts related to histidine kinases and sensor proteins found in the different treatments changed substantially among genes (Figure 6B). In general, transcription of most of the histidine kinases and sensor proteins (*CusS, BasS, BarA, EnvZ, FleS*, GAF, *arcB, PhoQ, CheA, SypF, YfhK, YfhA, CpxA, KdpD*, and *BaeS*) was more pronounced in the ammonium and organic matter treatments than in the other treatments. Some of these genes (*ChvG, CusS*, GAF, *BasS*) were dominant in the ammonium while very low or absent in the organic matter treatment.

## Discussion

### Effect of thiosulfate and ammonium on prokaryotic DIC fixation in the dark ocean

DIC fixation was not affected by thiosulfate, but its rates doubled in response to ammonium. The increase in DIC fixation rates in the ammonium treatment occurred without an increase in prokaryotic abundance or heterotrophic production rates, as indicated by a doubling in the DIC:PHP ratio as compared to the control (Table 2). Theses results point toward chemoautotrophic (ammonia oxidizing) prokaryotes, but not heterotrophs, as the main group responsible for the increased DIC fixation rates in the ammonium amendment. These results are consistent with the recent notion that prokaryotic DIC fixation is widespread among prokaryotes in the mesopelagic oceanic water column (Herndl et al., 2005; Kirchman et al., 2007; Varela et al., 2011). Based on the occurrence of *amoA* gene abundance, ammonia oxidation has been suggested as the main energy source for chemoautotrophic prokaryotes in the upper mesopelagic (Wuchter et al., 2006; Agogué et al., 2008; Beman et al., 2008). Thus, our results provide direct experimental support for ammonia as an important energy source for prokaryotic DIC fixation in the dark ocean.

### Effect of organic carbon on DIC fixation in the dark ocean: it's importance in deep-ocean carbon flux

Although dark DIC fixation has been commonly assumed to be of minor importance in oxygenated marine waters, several recent reports suggest that it might be in the same range as PHP in the deep Atlantic water column (Baltar et al., 2010b; Reinthaler et al., 2010). Our bulk (i.e., from the natural waters that the incubations originated from) DIC fixation rates were in the same range as PHP observed in those previous studies (i.e., *in situ* DIC:PHP ratio ranged between 0.2 and 0.7). In our experiments, DIC fixation and PHP increased by 1–2 orders of magnitude in the organic matter amendment (Figure 3). Remarkably, both these metabolic rates increased in the organic matter treatment, suggesting a potential link between the stimulation of auto- and hetero-trophic processes.

Several different metabolic processes can potentially contribute to heterotrophic DIC-fixation, such as anaplerotic reactions to replenish TCA cycle intermediates, synthesis of amino acids or nucleic acids precursors, and biosynthesis of fatty acids (Dijkhuizen and Harder, 1984; Erb, 2011). In fact, many ecologically relevant compounds are metabolized via “assimilatory carboxylases,” suggesting that these enzymes can be of importance for the global carbon cycle along with “autotrophic carboxylases” (Erb, 2011). Under oligotrophic conditions, anaplerotic DIC incorporation has been suggested to play an important role in compensating metabolic imbalances in marine Bacteria (González et al., 2008, 2011; Palovaara et al., 2014). It is also possible that if the hetetrotrophic metabolism of Bacteria is suddenly intensified (e.g., after an organic matter pulse), the DIC fixation rates also increase proportionally. This might depend on the availability of particular substrates, as anaplerotic reactions to replenish intermediates of the TCA cycle following synthesis of amino acids and nucleotides may also be enhanced when the extant organic precursors are not available to satisfy cellular requirements (Dijkhuizen and Harder, 1984). Considering the oligotrophic nature of the deep ocean and the sporadic, pulsed input of organic matter, our results suggest that anaplerotic reactions may at times contribute a larger proportion to dark DIC fixation than hitherto recognized.

Reinthaler et al. (2010) showed that DIC fixation in the dark ocean amounts to 15–53% of the phytoplankton export production. Baltar et al. (2010b) showed that 12–72% of the prokaryotic carbon demand required by heterotrophs in the mesopelagic ocean could be potentially supplied by deep-water prokaryotes via DIC fixation. Global rates of oceanic dark DIC fixation were estimated to amount to 0.77 Pg C y^−1^, indicating that this input of new organic carbon to the ocean is similar to that supplied by the world-rivers and that eventually buried in oceanic sediments (Middelburg, 2011). In the present work, we have shown that if organic carbon is supplied to the mesopelagic community, heterotrophic and autotrophic/mixotrophic activity increase concomitantly. Thus, our results suggest the potential of heterotrophic Bacteria to make substantial contributions to bulk DIC fixation—particularly under sporadic inputs of organic matter.

### Community changes and the potential ecological role of the “rare biosphere”

The strong increase in microbial activities in response to organic matter, including both PHP and DIC-fixation, was accompanied by large shifts in the prokaryotic community composition. A number of taxa abundant in the original waters, such as Archaea, some Bacteroidetes and Delta- and Gammaproteobacteria, declined substantially in abundance or completely disappeared during the course of the experiments. This was most pronounced in the organic matter treatment where a few Gammaproteobacteria rapidly increased and apparently outcompeted other prokaryotes, increasing from a relative abundance of ~6% in the natural waters to 72–95% of the prokaryotic community in the treatments (Figure 4, Tables S2, S3). Besides the general response by two Alteromonodales genera (*Alteromonas* and *Marinobacter*), a clear treatment-specific increase was found in the two different genera of Gammaproteobacteria *Vibrio* and *Pseudoalteromonas* and some Oceanospirillales (e.g., *Oleispira, Halomonas*) in the organic matter treatment. In experiments similar to ours with Artic surface seawater cultures, Alonso-Sáez et al. (2010) observed by microautoradiography combined with fluorescence *in situ* hybridization that the *Pseudoalteromonas-Colwellia* and *Oleispira* (i.e., Oceanospirillales) clusters were the most active of all prokaryotic groups in DIC uptake (ca. 30 and 70% of the active cells, respectively).

All of the prokaryotic groups attaining high relative abundances in the different treatments were members of the “rare biosphere” in the original waters. The most striking examples were *Vibrio* and *Pseudoalteromonas* in the organic matter treatment, increasing from a relative abundance of <0.5% to ~40 and >60%, respectively (Figure 4, Tables S2, S3). Although, different gamma-proteobacteria become the dominant members in response to organic carbon in the different experiments, DIC fixation increased in all experiments, suggesting some functional redundancy in DIC fixation rates across different gamma-proteobacteria members. The same heterotrophic Bacteria that were stimulated in our experiments (e.g., *Vibrio, Pseudoalteromonas, Marinobacter*, and *Halomonas*) are also frequently isolated from deep ocean environments (Raguénès et al., 1997; Teske et al., 2000; Kaye et al., 2004; Vetriani et al., 2005; Simon-Colin et al., 2008) although their ecology remains largely unknown. Some recent studies have found that such Bacteria can form sporadic blooms in natural seawater. (Alonso-Sáez et al., 2007) reported that *Glaciecola*, an Alteromonadaceae typically constituting <1% of the bacterial counts over a seasonal cycle in Blanes Bay, suddenly bloomed contributing up to 50% to total prokaryotic abundance. *Reinekea* spp. and *Formosa* spp., members of the rare biosphere in the original bacterial community, were reported to increase in relative abundance within 1 week to 16 and 24%, respectively, of the prokaryotic community in response to a diatom bloom in the North Sea (Teeling et al., 2012). Similarly, *Vibrio* populations have been reported to represent 54% of the sequences found during phytoplankton bloom conditions in seasonal studies in the English Channel, whereas this taxon was rare during the rest of the time series (i.e., abundance of 0–2%; Gilbert et al., 2012). Hence, it appears that under specific environmental conditions some bacteria of the “rare biosphere” attain high abundances, supporting the seed bank hypothesis of Pedrós-Alió (2006). There are limitations related to the realism of transferring incubation results to the natural environment that cannot be resolved at the moment (Stewart et al., 2012). Also, the choice of different organic matter substrates and concentrations might have changed the relative changes in DIC and PHP compared to natural situations. Bearing in mind these limitations, our experiments suggest that also in the mesopelagic realm a number of rare taxa are highly responsive to pulses of organic matter, and that such responses might affect the rates of autotrophic and heterotrophic prokaryotic activities.

### Gene expression sheds light on the potential importance and mechanisms of alternative CO_2_ fixation pathways and alkaline phosphatase in the dark ocean

The increase found in the relative proportion of *sox* transcripts in thiosulfate amended treatments indicated that, when given the opportunity, mesopelagic bacterioplankton in our region of study may activate their potential for utilization of reduced sulfur compounds as energy sources. Nevertheless, the upper mesopelagic prokaryotic community structure and their metabolic rates remained unaffected by the addition of thiosulfate, suggesting that thiosulfate oxidation was stimulated but not to an extent that affected C fixation. This parallels findings from experimental work with model microorganisms and field studies in mesopelagic waters, where cryptic sulfur cycling has been shown (Canfield et al., 2010; Durham et al., 2015).

The doubling in DIC fixation in the ammonium treatment coincided with very low relative abundance of transcripts of autotrophic DIC fixation enzymes (i.e., RuBisCO and propionyl-CoA carboxylase). Propionyl-CoA carboxylase has been suggested to be the main autotrophic CO_2_ fixation pathway linked to ammonia oxidation (Berg et al., 2010). Instead, a number of assimilatory carboxylases capable of DIC incorporation were highly abundant. It should be noted that the ammonia monooxygenase (*amoA*) expression in the ammonium amended treatment was similar to the controls and thiosulfate treatment, making it difficult to directly evaluate if ammonia oxidation through AmoA was the main mechanism responsible for the increase in DIC fixation in response to ammonium. Alternatively, or in addition, ammonia is known to be assimilated through glutamine synthesis which, in turn, increases dark CO_2_ fixation via PEP carboxykinase activity (Ohmori, 1981); the elevated expression of PEP carboxykinase as well as glutamine and glutamate synthases could point in this direction (Figure 6A), although these genes were fairly highly expressed in all treatments. We thus suggest that attention should be paid in future studies to distinguishing between complementary mechanisms through which ammonia is used for microbial DIC fixation in the upper mesopelagic realm.

Transcripts of some genes involved in DIC assimilation were generally present in the organic matter treatment. Phosphoenolpyruvate carboxykinase, phosphoenolpyruvate carboxylase, malic enzyme, and carbamoyl-phosphate synthase are common enzymes involved in anaplerotic reactions in heterotrophic organisms (Bar-Even et al., 2010; Erb, 2011). Phosphoenolpyruvate carboxykinase works as an anaplerotic reaction during amino acid synthesis, catalyzing the fixation of CO_2_ to phosphoenolpyruvate, whereas carbamoyl-phosphate synthase fixes CO_2_ to organic molecules during pyrimidine synthesis catalyzing the fixation of CO_2_ to phosphoenolpyruvate (Bolotin et al., 2004; Zamboni et al., 2004; Arioli et al., 2009). Recently, proteorhodopsin phototrophy in *Dokdonia* sp. MED134 was linked to carbon acquisition pathways via anaplerotic CO_2_ fixation, which provided up to one-third of the cell carbon (Palovaara et al., 2014). In a different study, high DIC fixation rates were correlated with PHP in dilution cultures with Arctic surface waters suggesting that most of the observed DIC fixation was due to heterotrophic Bacteria via fatty acids biosynthesis, anaplerotic pathways and/or leucine catabolism (Alonso-Sáez et al., 2010). Although these authors did not analyze gene expression data, they managed (via a clone library) to amplify sequences of genes involved in fatty acids biosynthesis, anaplerotic pathways and leucine catabolism, like acetyl-CoA carboxylases and carbamoyl phosphate synthase. This is consistent with the increase we found in the expression level of genes involved in heterotrophic/mixotrophic DIC fixation. These results highlight the potential of heterotrophic prokaryotes to carry out significant levels of DIC fixation in response to inputs of organic matter via fatty acids biosynthesis, anaplerotic pathways and leucine catabolism enzymes.

APase activity is supposed to be regulated by the concentration of its end-product, decreasing with increasing phosphate concentrations (Chrost, 1991). Since phosphate is readily available in the deep ocean, APase activity would be expected to be low. However, high APase activities at high phosphate concentrations have been found in the deep Indian and Atlantic Ocean, leading to the paradox of high alkaline phosphatase expression at high end-product concentrations (Hoppe and Ullrich, 1999; Baltar et al., 2009b). In our experiments, APase most pronouncedly increased in the organic matter (in 3 experiments) and the ammonium treatment (in Expt. 2). This is surprising since phosphate was readily available in our deep-water samples (Table 1). Focusing on the Expt. 2 gene expression analysis, the relative abundance of the Pho regulon—a global regulatory mechanism involved in bacterial phosphate management including genes encoding alkaline phosphatases—was found to be upregulated in the ammonium and organic matter treatment. However, in the organic matter treatment all measured rates also increased, whereas in the ammonium amendment only dark CO_2_ fixation and APase rates were enhanced. One possible explanation for the up-regulation of APase rates and the genes involved could be due to the consumption of the available phosphate during the incubation. Although, we do not completely discard this possibility, the lack of an increase in the prokaryotic abundance or heterotrophic activity in the ammonium treatment suggests that phosphate was not been extensively consumed. Moreover, doing a back of the envelop calculation, assuming a P quota of 0.78 fg/cell, the phosphate needed to account for the PA biomass increase during OM treatment in Expt 2 (where the biggest PA change occurred) would be around 0.27 μM, which would reduce the P concentration in Expt 2 from 1.77 to 1.55 μM. This concentration would be above what is considered limiting in the sense of triggering APase activity.

Several studies have also reported phosphate and carbon cross-talk, and the regulation of Pho regulon members by different carbon sources and regulators (reviewed in Santos-Beneit, 2015). The global cAMP-dependent transcriptional regulator (GlxR) in *C. glutamicum* binds to the *pstS* promoter and activates its transcription under phosphate limitation in a carbon source-dependent manner (Panhorst et al., 2011). Other studies have found additional pho-dependent promoters controlled by the carbon source in *pstS* in *S. coelicolor* (Díaz et al., 2005; Esteban et al., 2008; Santos-Beneit et al., 2009). Based on the strong up-regulation observed in *S. lividans* of the *pstS* gene in the presence of fructose and other carbon sources, C and P metabolism were suggested to be linked in the regulation of genes (Díaz et al., 2005). These authors concluded that the activation of carbohydrate metabolism in general, produced by an excess availability of organic C sources in the media, produced an intense decrease in intracellular phosphate and an activation of the Pho regulon. *PstS* expression is triggered in order to capture phosphate from the environment in response to the requirement of extra phosphate to phosphorylate the high concentration of internalized sugar (Díaz et al., 2005). The fact that *pstS* were strongly up-regulated in response to organic carbon (the organic matter treatment accounted for >50% of the *pstS* transcripts found in all treatments, Figure 6B), suggests that this C-P cross talk might have occurred also in our study. Thus, the activation of the Pho regulon seemed to be related to cross-activation by nonpartner histidine kinases, and/or the activation of genes involved in the regulation of the elemental balance during catabolic processes. Increased C or N bioavailability thus appears to elicit a phosphate deficiency inside cells and activate the Pho regulon. These results suggest that pulses of material with different C or N content or different elemental ratios can activate APase irrespectively of the environmental phosphate concentration.

Bacterial pathways involved in C, N, and P metabolisms must be coordinated in a regulated manner, but the mechanisms involved in such interactions are just starting to be understood (Santos-Beneit, 2015). Two-component systems, with a histidine kinase and a partner response regulator, are the predominant genetic regulatory mechanisms in bacteria. Different histidine kinases are autophosphorylated in response to particular environmental signals, which then act as phosphoryl donors for autophosphorylation of their partner response regulators. However, nonpartner histidine kinases and a partner response regulators also interact causing cross-regulations among two-component systems (Wanner, 1992). The PhoR (histidine kinase)/PhoB (response regulator) two-component systems control the Pho regulon. Several cross-talks among two-component systems have been reported, at least seven different kinases are able to phosphorylate PhoB in *E. coli*, including *VanS, ArcB, CreC, kdpD, QseC, BaeS*, and *EnvZ* (Amemura et al., 1990; Fisher et al., 1995; Zhou et al., 2005). In our study, most of the transcripts of histidine kinases genes were found in the ammonium and the organic matter treatment (Figure 6B), as well as the highest APase rates (Figure 3), and alkaline phosphatase gene transcripts (around 30% for ammonium and organic matter treatments compared to around 20% for the thiosulfate and the control treatment). The proportion of transcripts of some histidine kinases genes was also elevated in the ammonium treatment (e.g., *ChvG, CusS*, and GAF), in the organic matter (e.g., *BaeS, kdpD, and CpXA*), or similarly prevalent in both the organic matter and the ammonium treatment (e.g., *BarA, ArcB, and FleS*). This potentially indicates that some histidine kinases might be related to the activation of APase.

The two-component CbrA-CbrB system controls the expression of several catabolic pathways in response to different C:N ratios and ensures the intracellular C:N balance in *P. aeruginosa* (Nishijyo et al., 2001). High ammonia concentrations favor dephosphorylation of NtrC and, hence, loss of activation of σ^54^-dependent promoters (Ninfa et al., 1995). Since amino acid catabolism is mainly controlled by the NtrB-NtrC system and σ^54^-RNA polymerase, an excess of ammonia tends to switch off this catabolic pathways (Nishijyo et al., 2001). These authors suggested that CbrB prevents this effect of excess ammonia enabling the functioning of catabolic pathways under different C:N conditions. In our experiment, most of the transcripts of CbrA and CbrB were found in the ammonium amended treatment (Figure 6A), suggesting that the microbial community was responding to the sudden supply of ammonium to balance the C:N ratio and an interaction between C and N regulation.

## Conclusions

Taken together, our results indicate that the metabolic activity of the prokaryotic community of the mesopelagic subtropical North Atlantic is not stimulated by thiosulfate (unless cryptically), but by amendments with ammonium and organic carbon. Elevated dark DIC fixation was observed in both ammonium and organic carbon amendments linked to autotrophic and anaplerotic pathways, respectively. An activation of the Pho regulon was observed in response to ammonium and organic carbon probably related to cross-activation by nonpartner histidine kinases, and/or the activation of genes involved in the regulation of elemental balance during catabolic processes. Only the addition of organic carbon compounds (but not in thiosulfate and ammonium) altered the prokaryotic community composition, stimulating ‘rare biosphere’ members. Thus, episodic events such as sedimentation of organic matter into the mesopelagic zone might trigger the rapid increase of originally rare members of the prokaryotic community, affecting N and P cycling and enhancing heterotrophic/mixotrophic and autotrophic carbon assimilation rates, and ultimately affecting the dark ocean's carbon fluxes. Our work, based on experimental manipulation, discovered and analyzed processes and mechanisms that could be important in the dark ocean, which would require novel efforts *in situ* to uncover their true importance.

## Author contributions

FB, GH, and JaP designed research. FB, DL, JoP, TR, and IL collected data and all authors performed parts of the data analysis. FB wrote the paper with contributions from all authors.

## Funding

This research was supported by grants from the Crafoord Foundation and the Swedish Research Council to JaP. FB was supported by an University of Otago Research Grant. GH was supported by the ESF MOCA project and the Austrian Science Fund (FWF) projects: I486-B09 and P23234-B11. TR was supported by the Austrian Science Fund (FWF) project PADOM (P23221-B11).

### Conflict of interest statement

The authors declare that the research was conducted in the absence of any commercial or financial relationships that could be construed as a potential conflict of interest.
