# Supplementary material for: Prokaryotic Responses to Ammonium and Organic Carbon Reveal Alternative CO2 Fixation Pathways and Importance of Alkaline Phosphatase in the Mesopelagic North Atlantic
Source: Front Microbiol. 2016 Oct 21;7:1670. doi: 10.3389/fmicb.2016.01670 (PMC5073097; doi:10.3389/fmicb.2016.01670)
Supplement: Supplementary file 1 [file DataSheet1.PDF]

Supplementary Material

Prokaryotic responses to nutrients reveal alternative CO_2 fixation pathways and importance of alkaline phosphatase in the mesopelagic North Atlantic

Federico Baltar, Daniel Lundin, Joakim Palovaara, Itziar Lekunberri, Thomas Reinthaler, Gerhard J. Herndl, Jarone Pinhassi

1 Supplementary Tables

Table S1. Relative transcript abundances (counts per million) retrieved from the different treatments for different gene categories, followed by the ratios.

seed1	C	TS	AM	OM		TS	AM	OM
Clustering-based subsystems	218663.0	162019.0	185065.0	203548.0		0.7	0.8	0.9
Not in SEED	171084.0	198098.0	225647.0	153694.0		1.2	1.3	0.9
Carbohydrates	187441.0	202882.0	121399.0	139731.0		1.1	0.6	0.7
Protein Metabolism	122076.0	110810.0	170446.0	238117.0		0.9	1.4	2.0
Amino Acids and Derivatives	64631.0	69631.0	88506.0	93499.0		1.1	1.4	1.4
Miscellaneous	70464.0	68236.0	73692.0	86438.0		1.0	1.0	1.2
Fatty Acids, Lipids, and Isoprenoids	99775.0	51741.0	49268.0	32189.0		0.5	0.5	0.3
RNA Metabolism	46173.0	45809.0	56533.0	64109.0		1.0	1.2	1.4
Cofactors, Vitamins, Prosthetic Groups, Pigments	44017.0	48821.0	55934.0	60544.0		1.1	1.3	1.4
Virulence, Disease and Defense	73549.0	66617.0	41204.0	20474.0		0.9	0.6	0.3
Membrane Transport	38070.0	49362.0	59933.0	49707.0		1.3	1.6	1.3
Respiration	34186.0	38801.0	52864.0	46520.0		1.1	1.5	1.4
Stress Response	37417.0	34209.0	45560.0	47141.0		0.9	1.2	1.3
Cell Wall and Capsule	26146.0	27943.0	36052.0	34944.0		1.1	1.4	1.3
Motility and Chemotaxis	19356.0	19199.0	26916.0	36397.0		1.0	1.4	1.9
Nitrogen Metabolism	31347.0	37514.0	11619.0	20379.0		1.2	0.4	0.7
Metabolism of Aromatic Compounds	16447.0	20440.0	33938.0	20959.0		1.2	2.1	1.3
DNA Metabolism	19616.0	22448.0	21795.0	22269.0		1.1	1.1	1.1
Nucleosides and Nucleotides	18344.0	19192.0	20783.0	26039.0		1.0	1.1	1.4
Phages, Prophages, Transposable elements, Plasmids	16910.0	18266.0	13972.0	11684.0		1.1	0.8	0.7
Regulation and Cell signaling	13136.0	15832.0	15497.0	15097.0		1.2	1.2	1.1
Cell Division and Cell Cycle	12206.0	10002.0	14068.0	18313.0		0.8	1.2	1.5
Iron acquisition and metabolism	8504.0	10703.0	15819.0	13977.0		1.3	1.9	1.6
Sulfur Metabolism	15128.0	13294.0	9927.0	8110.0		0.9	0.7	0.5
Phosphorus Metabolism	9879.0	8087.0	7953.0	7928.0		0.8	0.8	0.8
Potassium metabolism	2388.0	2743.0	4293.0	2749.0		1.1	1.8	1.2
Secondary Metabolism	1580.0	2177.0	2341.0	952.0		1.4	1.5	0.6
Dormancy and Sporulation	627.0	800.0	1309.0	1382.0		1.3	2.1	2.2
Photosynthesis	463.0	557.0	792.0	542.0		1.2	1.7	1.2

Table S2. Relative abundance (%) of sequences obtained at the end of Expt. 1. N: original waters, C: unamended control, TS: thiosulfate, AM: ammonium, OM: acetate + pyruvate, , a, b – denote replicate treatments, - indicates not detected.

**Taxon**	**N1**	**C1a**	**C1b**	**TS1b**	**AM1a**	**AM1b**	**OM1a**	**OM1b**
Archaea Euryarchaeota	1.171	-	-	-	-	-	-	-
Archaea; Thaumarchaeota	46.854	2.568	3.175	3.146	0.351	0.501	-	-
Bacteria;Acidobacteria;Acidobacteria;Acidobacteriales;Acidobacteriaceae;Gp21	0.022	-	-	-	-	-	-	-
Bacteria;Acidobacteria;Acidobacteria;Acidobacteriales;Acidobacteriaceae;Gp26	0.080	-	-	-	-	-	-	-
Bacteria;Acidobacteria;Acidobacteria;Acidobacteriales;Acidobacteriaceae;Gp6	0.133	-	-	-	-	-	-	-
Bacteria;Actinobacteria;Actinobacteria;Actinobacteridae;Actinomycetales;Corynebacterineae;Mycobacteriaceae	0.018	-	-	-	-	-	-	-
Bacteria;Actinobacteria;Actinobacteria;Actinobacteridae;Actinomycetales;Other	0.612	0.003	-	-	-	-	-	-
Bacteria;Actinobacteria;Actinobacteria;Other	1.849	0.012	-	-	-	-	-	-
Bacteria;Bacteroidetes;Flavobacteria;Flavobacteriales;Cryomorphaceae;Other	0.124	-	-	-	-	-	-	-
Bacteria;Bacteroidetes;Flavobacteria;Flavobacteriales;Flavobacteriaceae;Bizionia	0.004	-	-	-	-	-	0.278	1.905
Bacteria;Bacteroidetes;Flavobacteria;Flavobacteriales;Flavobacteriaceae;Other	0.426	0.015	-	-	-	-	2.782	-
Bacteria;Bacteroidetes;Flavobacteria;Flavobacteriales;Flavobacteriaceae;Tenacibaculum	0.004	0.009	-	-	-	-	-	-
Bacteria;Bacteroidetes;Flavobacteria;Flavobacteriales;Other	0.018	-	-	-	-	-	-	-
Bacteria;Bacteroidetes;Other	0.191	0.009	-	-	-	-	-	0.476
Bacteria;Bacteroidetes;Sphingobacteria;Sphingobacteriales;Flexibacteraceae	0.106	0.018	-	-	-	-	-	-
Bacteria;Bacteroidetes;Sphingobacteria;Sphingobacteriales;Other	0.009	-	-	-	-	-	-	-
Bacteria;Chlamydiae;Chlamydiae;Chlamydiales	0.004	-	-	-	-	-	-	-
Bacteria;Chloroflexi;Anaerolineae	0.004	-	-	-	-	-	-	-
Bacteria;Cyanobacteria;Cyanobacteria;FamilyII;GpIIa	0.009	-	-	-	-	-	-	-
Bacteria;Firmicutes	0.004	-	-	-	-	-	-	-
Bacteria;Lentisphaerae;Lentisphaerae;Lentisphaerales;Lentisphaeraceae;Lentisphaera	0.004	-	-	-	-	-	-	-
Bacteria;Nitrospira;Nitrospira;Nitrospirales;Nitrospiraceae;Nitrospira	0.009	-	-	-	-	-	-	-
Bacteria; Other	19.307	2.366	0.719	1.655	1.311	2.163	2.643	2.857

Bacteria;Proteobacteria;Alphaproteobacteria;Rhodobacterales;Rhodobacteraceae	0.049	1.754	0.781	2.483	2.712	8.667	2.225	11.429
Bacteria;Proteobacteria;Deltaproteobacteria;Desulfobacterales;Nitrospinaceae;Nitrospina	0.102	-	-	-	-	-	-	-
Bacteria;Proteobacteria;Deltaproteobacteria; SAR324	7.207	0.051-	-	-	-	0.167-	-	-
Bacteria;Proteobacteria;Gammaproteobacteria;Alteromonadales;Alteromonadaceae;Alteromonas	0.044	25.015	24.219	18.046	30.971	24.167	1.252	0.952
Bacteria;Proteobacteria;Gammaproteobacteria;Alteromonadales;Incertae sedis7;Marinobacter	0.018	63.806	66.406	72.020	60.717	59.167	42.698	38.571
Bacteria;Proteobacteria;Gammaproteobacteria;Alteromonadales;Other	0.013	0.656	-	0.497	1.225	0.167	0.278	-
Bacteria;Proteobacteria;Gammaproteobacteria;Alteromonadales;Pseudoalteromonadaceae;Pseudoalteromonas	0.049	0.042	-	0.166	-	-	0.139	0.476
Bacteria;Proteobacteria;Gammaproteobacteria;Legionellales;Coxiellaceae;Coxiella	0.013	-	-	-	-	-	-	-
Bacteria;Proteobacteria;Gammaproteobacteria;Oceanospirillales;Alcanivoraceae;Alcanivorax	0.013	0.972	0.781	0.331	0.262	2.333	-	0.952
Bacteria;Proteobacteria;Gammaproteobacteria;Oceanospirillales;Oceanospirillaceae;Marinomonas	0.013	-	-	-	-	-	3.477	1.429
Bacteria;Proteobacteria;Gammaproteobacteria;Oceanospirillales;Oceanospirillaceae;Oleispira	0.044	0.048	-	0.166	-	-	0.139	0.476
Bacteria;Proteobacteria;Gammaproteobacteria;Oceanospirillales;Oceanospirillaceae;Other	0.013	0.597	0.781	-	0.175	-	0.139	-
Bacteria;Proteobacteria;Gammaproteobacteria;Oceanospirillales;Other	0.004	-	-	-	-	-	-	-
Bacteria;Proteobacteria;Gammaproteobacteria;Other	5.246	1.056	2.344	0.993	1.925	2.000	2.921	1.429
Bacteria;Proteobacteria;Gammaproteobacteria;SAR156	1.005	0.009	-	-	0.088	-	-	-
Bacteria;Proteobacteria;Gammaproteobacteria;Thiotrichales	0.009	-	-	-	-	-	-	-
Bacteria;Proteobacteria;Gammaproteobacteria;Vibrionales;Vibrionaceae;Other	-	-	-	-	-	-	1.808	1.905
Bacteria;Proteobacteria;Gammaproteobacteria;Vibrionales;Vibrionaceae;Photobacterium	0.004	-	-	-	-	-	-	-
Bacteria;Proteobacteria;Gammaproteobacteria;Vibrionales;Vibrionaceae;Vibrio	0.027	0.012	-	-	-	-	39.221	37.143
Bacteria;Verrucomicrobia;Verrucomicrobiae;Verrucomicrobiales;Opitutaceae;Opitutus	0.133	-	-	0.166	-	-	-	-
Bacteria;Verrucomicrobia;Verrucomicrobiae;Verrucomicrobiales;Other	0.089	-	-	-	-	-	-	-
Bacteria;Verrucomicrobia;Verrucomicrobiae;Verrucomicrobiales;Verrucomicrobiaceae	0.177	-	-	-	-	-	-	-

Table S3. Relative abundance (%) of sequences obtained at the end of Expt. 3. N: original waters, C: unamended control, TS: thiosulfate, AM: ammonium, OM: acetate + pyruvate. a, b – denote replicate treatments, - indicates not detected

**Taxon**	**N3**	**C3a**	**C3b**	**TS3a**	**TS3b**	**AM3a**	**AM3b**	**OM3a**	**OM3b**
Archaea;Crenarchaeota;Thermoprotei	0.024	-	-	0.007	0.010	0.009	0.003	-	-
Archaea;Euryarchaeota	0.875	0.050	0.428	0.165	0.822	0.359	0.401	0.004	-
Archaea;Thaumarchaeota	44.530	10.948	7.803	12.124	4.006	9.101	8.970	0.632	0.237
Bacteria;Acidobacteria;Acidobacteria;Acidobacteriales;Acidobacteriaceae;Gp21	0.020	0.010	-	0.011	0.010	0.004	0.003	-	-
Bacteria;Acidobacteria;Acidobacteria;Acidobacteriales;Acidobacteriaceae;Gp26	0.049	0.010	0.053	0.004	0.017	0.004	-	0.004	-
Bacteria;Acidobacteria;Acidobacteria;Acidobacteriales;Acidobacteriaceae;Gp6	0.171	0.040	0.053	0.025	0.050	0.022	0.013	0.017	-
Bacteria;Actinobacteria;Actinobacteria;Actinobacteridae;Actinomycetales;Corynebacterineae;Mycobacteriaceae	0.010	-	-	-	-	0.004	-	-	-
Bacteria;Actinobacteria;Actinobacteria;Actinobacteridae;Actinomycetales;Other	0.459	0.159	0.107	0.093	0.316	0.013	0.029	-	-
Bacteria;Actinobacteria;Actinobacteria;Other	1.495	0.357	0.641	0.397	0.912	0.276	0.270	0.013	-
Bacteria;Bacteroidetes;Flavobacteria;Flavobacteriales;Cryomorphaceae	0.068	0.050	-	0.050	0.070	0.022	0.036	-	-
Bacteria;Bacteroidetes;Flavobacteria;Flavobacteriales;Flavobacteriaceae;Other	0.127	0.030	0.107	0.107	0.047	0.018	0.039	0.009	0.004
Bacteria;Bacteroidetes;Flavobacteria;Flavobacteriales;Flavobacteriaceae;Tenacibaculum	0.005	-	-	0.004	-	-	-	-	-
Bacteria;Bacteroidetes;Other	0.137	0.119	-	0.029	0.103	0.026	0.026	-	-
Bacteria;Bacteroidetes;Sphingobacteria;Sphingobacteriales;Flexibacteraceae	0.103	0.050	-	0.032	0.067	-	0.016	-	-
Bacteria;Chlamydiae;Chlamydiae;Chlamydiales	0.015	-	-	-	-	-	-	-	-
Bacteria;Chloroflexi;Anaerolineae	0.044	-	-	-	-	-	-	-	-
Bacteria;Other	19.033	7.456	7.765	7.129	12.810	3.686	4.575	1.360	0.744
Bacteria;Planctomycetes;Planctomycetacia;Planctomycetales;Planctomycetaceae;Blastopirellula	0.029	-	-	-	-	-	-	-	-
Bacteria;Planctomycetes;Planctomycetacia;Planctomycetales;Planctomycetaceae;Other	0.435	0.050	-	0.007	0.060	-	0.013	0.004	0.004
Bacteria;Planctomycetes;Planctomycetacia;Planctomycetales;Planctomycetaceae;Planctomyces	0.078	-	-	0.007	0.003	-	-	-	-
Bacteria;Proteobacteria;Alphaproteobacteria;Other	0.767	0.169	0.428	0.182	0.366	0.088	0.065	0.030	0.033
Bacteria;Proteobacteria;Alphaproteobacteria;Rhodobacterales;Rhodobacteraceae;Other	0.044	1.061	1.015	3.723	2.904	1.764	1.990	9.966	4.859
Bacteria;Proteobacteria;Alphaproteobacteria; Rickettsiales;SAR11	16.332	3.511	3.546	3.731	2.816	1.615	2.665	0.073	0.007
Bacteria;Proteobacteria;Alphaproteobacteria;Rhodobacterales;Rhodobacteraceae;Sulfitobacter	0.005	-	-	0.004	0.003	-	0.003	-	0.007
Bacteria;Proteobacteria;Deltaproteobacteria;Desulfobacterales;Nitrospinaceae;Nitrospina	0.108	0.030	-	0.011	0.037	0.009	0.007	-	-
Bacteria;Proteobacteria;Deltaproteobacteria; SAR324	7.545	0.569	1.075	0.567	0.738	0.263	0.330	0.030	0.007
Bacteria;Proteobacteria;Gammaproteobacteria;Alteromonadales;Alteromonadaceae;Alteromonas	0.454	5.405	23.570	6.552	7.579	10.305	8.533	7.800	0.657
Bacteria;Proteobacteria;Gammaproteobacteria;Alteromonadales;Alteromonadaceae;Other	0.015	0.010	0.053	0.032	0.027	0.013	0.010	0.004	0.004

Bacteria;Proteobacteria;Gammaproteobacteria;Alteromonadales;Colwelliaceae;Colwellia	0.010	0.426	0.534	0.579	0.613	0.350	0.453	0.021	0.007
Bacteria;Proteobacteria;Gammaproteobacteria;Alteromonadales;Colwelliaceae;Other	0.010	0.397	0.428	0.501	0.433	0.114	0.176	0.004	-
Bacteria;Proteobacteria;Gammaproteobacteria;Alteromonadales;Idiomarinaceae;Idiomarina	0.010	-	-	-	-	-	-	-	-
Bacteria;Proteobacteria;Gammaproteobacteria;Alteromonadales;Incertae sedis7;Marinobacter	0.098	40.797	28.274	42.395	34.108	53.426	53.904	12.644	3.710
Bacteria;Proteobacteria;Gammaproteobacteria;Alteromonadales;Other	0.034	0.407	0.695	0.658	0.443	0.530	0.495	1.149	0.354
Bacteria;Proteobacteria;Gammaproteobacteria;Alteromonadales;Pseudoalteromonadaceae;Pseudoalteromonas	0.337	0.387	1.336	1.280	0.706	0.722	0.485	57.800	85.454
Bacteria;Proteobacteria;Gammaproteobacteria;Legionellales;Coxiellaceae;Coxiella	0.029	-	-	-	-	-	0.003	-	-
Bacteria;Proteobacteria;Gammaproteobacteria;Oceanospirillales;Alcanivoraceae;Alcanivorax	0.029	2.221	2.672	2.750	3.849	3.498	3.342	0.393	0.085
Bacteria;Proteobacteria;Gammaproteobacteria;Oceanospirillales;Halomonadaceae;Halomonas	0.054	-	-	0.011	0.017	0.013	0.003	0.384	0.222
Bacteria;Proteobacteria;Gammaproteobacteria;Oceanospirillales;Oceanospirillaceae;Marinomonas	0.010	0.089	0.160	0.064	0.033	0.385	0.016	4.024	0.447
Bacteria;Proteobacteria;Gammaproteobacteria;Oceanospirillales;Oceanospirillaceae;Oleispira	0.020	18.833	12.520	11.034	18.348	9.223	9.002	0.265	0.011
Bacteria;Proteobacteria;Gammaproteobacteria;Oceanospirillales;Other	0.015	0.060	-	0.118	0.113	0.070	0.072	-	-
Bacteria;Proteobacteria;Gammaproteobacteria;Other	5.023	5.801	5.986	5.257	6.630	3.782	3.654	3.281	1.547
Bacteria;Proteobacteria;Gammaproteobacteria;SAR156	1.069	0.399	0.591	0.316	0.781	0.224	0.340	-	-
Bacteria;Proteobacteria;Gammaproteobacteria;Thiotrichales	0.010	0.010	-	0.004	0.037	0.004	0.013	-	-
Bacteria;Proteobacteria;Gammaproteobacteria;Vibrionales;Vibrionaceae;Other	0.005	-	-	0.004	0.003	-	0.007	-	0.343
Bacteria;Proteobacteria;Gammaproteobacteria;Vibrionales;Vibrionaceae;Photobacterium	0.010	-	-	0.004	0.003	-	-	-	0.004
Bacteria;Proteobacteria;Gammaproteobacteria;Vibrionales;Vibrionaceae;Vibrio	0.024	0.020	0.107	0.007	0.013	0.022	0.016	0.085	1.252
Bacteria;Verrucomicrobia;Verrucomicrobiae;Verrucomicrobiales;Opitutaceae;Opitutus;Other	0.103	0.069	0.053	0.021	0.083	0.026	0.016	0.004	-
Bacteria;Verrucomicrobia;Verrucomicrobiae;Verrucomicrobiales;Other;Other;Other	0.073	-	-	-	0.007	0.009	0.003	-	-
Bacteria;Verrucomicrobia;Verrucomicrobiae;Verrucomicrobiales;Verrucomicrobiaceae	0.049	-	-	0.004	0.007	-	0.003	-	-
